# Optimal Combination of Mixing Units Using the Design of Experiments Method

**DOI:** 10.3390/mi12080985

**Published:** 2021-08-19

**Authors:** Makhsuda Juraeva, Dong-Jin Kang

**Affiliations:** School of Mechanical Engineering, Yeungnam University, Gyoungsan 712-749, Korea; 20101943@edms.yu.ac.kr

**Keywords:** degree of mixing, statistical significance, mixing cell with baffles (MC-B), cross-channel SAR (CC-SAR), combination scheme

## Abstract

A passive micromixer was designed by combining two mixing units: the cross-channel split and recombined (CC-SAR) and a mixing cell with baffles (MC-B). The passive micromixer was comprised of eight mixing slots that corresponded to four combination units; two mixing slots were grouped as one combination unit. The combination of the two mixing units was based on four combination schemes: (A) first mixing unit, (B) first combination unit, (C) first combination module, and (D) second combination module. The statistical significance of the four combination schemes was analyzed using analysis of variance (ANOVA) in terms of the degree of mixing (DOM) and mixing energy cost (MEC). The DOM and MEC were simulated numerically for three Reynolds numbers (Re = 0.5, 2, and 50), representing three mixing regimes. The combination scheme (B), using different mixing units in the first two mixing slots, was significant for Re = 2 and 50. The four combination schemes had little effect on the mixing performance of a passive micromixer operating in the mixing regime of molecular dominance. The combination scheme (B) was generalized to arbitrary mixing slots, and its significance was analyzed for Re = 2 and 50. The general combination scheme meant two different mixing units in two consecutive mixing slots. The numerical simulation results showed that the general combination scheme was statistically significant in the first three combination units for Re = 2, and significant in the first two combination units for Re = 50. The combined micromixer based on the general combination scheme throughout the entire micromixer showed the best mixing performance over a wide range of Reynolds numbers, compared to other micromixers that did not adopt completely the general combination scheme. The most significant enhancement due to the general combination scheme was observed in the transition mixing scheme and was negligible in the molecular dominance scheme. The combination order was less significant after three combination units.

## 1. Introduction

Mixing is one of the most common phenomena encountered in chemical processes and biological science. Micromixers are used widely to improve the mixing of two fluids in microfluidic devices for microreactors and micro-total analysis systems. On the other hand, the microdimensions of microfluidic devices and their slow fluid velocity lead to very low Reynolds number flows, and mixing is dominated by molecular diffusion. Therefore, mixing enhancement is a crucial design goal of micromixers [1].

Micromixers can be classified into two categories: active and passive micromixers. Active micromixers use an external energy source to promote mixing, and the corresponding structure is relatively more complicated than passive micromixers. Various energy sources are used for active micromixers, e.g., acoustic [2], magnetic [3], electric [4], thermal [5], and pressure [6]. On the other hand, most passive micromixers are based on the geometric structure of micromixers to enhance mixing. The geometric structures of passive micromixers are usually aimed at promoting chaotic advection of mixing fluids. Therefore, passive micromixers have no moving parts from external energy sources and are much simpler to integrate into microfluidic systems. Diverse design concepts for passive micromixers have been reported. Some examples include: micromixers with a staggered herringbone [7], repeated surface groove [8], twisted channel wall [9], a block at the junction [10], manifold junction [11], baffles [12], serpentine channel [13], and split-and-recombine (SAR) [14].

Among the various design concepts for passive micromixers, the process of combining different mixing units is a new approach in the design of passive micromixers. For example, Raza et al. [15] proposed the unbalanced SAR micromixer combined with curved channels with baffles and reported greatly improved mixing performance over earlier SAR micromixers. Bazaz et al. [16] proposed a micromixer that combined various mixing units, such as teardrop, obstruction, nozzle & pillar, and Tesla. They showed that a combination of planar mixing units was a useful strategy to build high-performance micromixers. Li et al. [17] combined a planar asymmetric SAR with dislocated subchannels. They reported that their combined micromixer improved mixing because of the multi-directional vortices formed in the dislocated subchannels. Makhsuda et al. [18] combined a cross-channel SAR (CC-SAR) with a mixing cell with baffles (MC-B) and reported improved mixing performance over a wide range of flow rates. They showed that the CC-SAR and MC-B were useful mixing units when building a high-performance passive micromixer.

Several studies have examined the optimization of combined micromixers. For example, Hossain et al. [19] optimized a micromixer with staggered herringbone grooves on the top and bottom walls using the mixing index and friction factor as objective functions. Yoshimura et al. [20] used a topology optimization method with a surrogate-assisted genetic algorithm and applied it to a staggered herringbone micromixer. They evaluated how the combination of grooves with different geometric aspects and the number of grooves affected the mixing performance. Solehati et al. [21] used the Taguchi method to optimize a wavy channel micromixer. They used pumping power and figure of merit as objective functions and reported that the Taguchi method was robust in its ability to determine the optimal combination of design parameters.

For a given number of mixing units, various combinations are possible when designing a combined micromixer. The combination scheme used to design a combined micromixer is assumed to affect the mixing performance. Although various studies were carried out to optimize combined micromixers, the combination scheme of mixing units has not been studied. Most research on the optimization of combined micromixers focused on determining the geometric design parameters for a given combination scheme of mixing units. The physical meaning and reasons for specific combination schemes were not dealt with in detail.

The mixing of a micromixer is usually divided into three regimes: molecular diffusion dominance, transition, and chaotic advection dominance mixing [15,18]. Considering the mixing regimes, the cross-channel SAR (CC-SAR) and the mixing cell with baffles (MC-B) were chosen as basic mixing units. In previous research, they showed quite different flow and mixing characteristics [18]. The CC-SAR generates a Dean vortex in the streamwise direction, and its mixing performance is better than the MC-B for Reynolds numbers larger than about 50. The CC-SAR also performs better in the mixing regime of chaotic advection dominance. On the contrary, the MC-B generates vortex flow in the transverse direction and leads to mixing enhancement, especially in the mixing regime of molecular diffusion dominance. However, the MC-B requires a larger pressure load than the CC-SAR. Therefore, a proper combination of the CC-SAR and MC-B can be expected to perform better than that of a passive micromixer based on either of the CC-SAR or the MC-B alone. In theory, there are many possible combinations, based on the two mixing units. The question is: what kind of combination scheme should be used? Its statistical significance is an important design advantage that could help to obtain a higher DOM.

This paper proposed four combination schemes to construct a combined micromixer with two mixing units: CC-SAR and MC-B. The combined micromixer had eight slots to house two different mixing units. The statistical significance of each combination scheme was studied using the design of experiments method, and an optimal combination of two mixing units was proposed based on the results of analysis of variance (ANOVA). In addition, the statistical significance of combination schemes was evaluated based on the degree of mixing (DOM) and mixing energy cost (MEC).

The mixing performance of a combined micromixer was simulated numerically using the commercial software, Ansys^®^ Fluent 19.2 [22]. Numerical simulations were carried out for three Reynolds numbers (Re = 0.5, 2, and 50) to evaluate the statistical significance of the four combination schemes. The three Reynolds numbers, Re = 0.5, 2, and 50, represented three mixing regimes in which the dominant mixing mechanisms differed. For Re = 0.5, molecular diffusion was the major mixing mechanism, while chaotic advection played a significant role for Re = 50. Re = 2 represented the regime where mixing due to the molecular diffusion and chaotic advection were of equal significance.

## 2. Combined Micromixers and Combination Schemes

**Figure 1:**
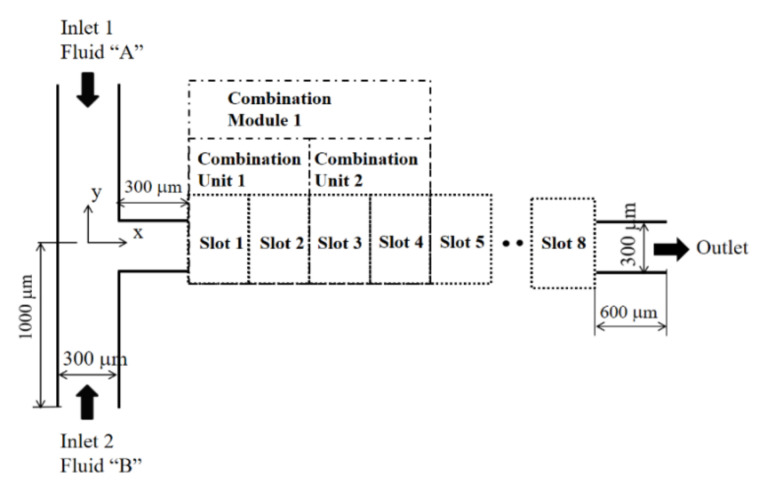
Diagram of a combined micromixer with eight slots (nonproportional).

**Figure 2:**
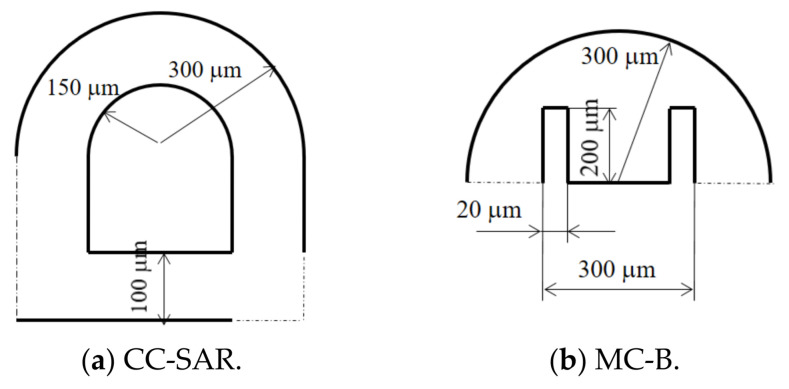
Two mixing units (nonproportional).

Figure 1 shows the layout of a combined micromixer with eight slots. Any of the two mixing units could be placed in each slot (CC-SAR and MC-B). The cross-section of the inlets and outlet was a rectangle, 300 μm wide and 120 μm deep. Both inlets 1 and 2 were 1000 μm long. Figure 2 shows the two mixing units used to construct a combined micromixer.

The eight slots of the combined micromixer were defined; two consecutive slots formed a combination unit, and two combination units formed a combination module. Therefore, a combined micromixer was constructed by choosing two combination modules. Four combination schemes were used to construct a combined micromixer with two mixing units, as follows:

(A) First slot: CC-SAR or MC-B.

(B) First combination unit: same mixing units or different mixing units.

(C) First combination module: Same combination units or reversed combination units.

(D) Second combination module: same combination module or reversed combination module.

**Figure 3:**
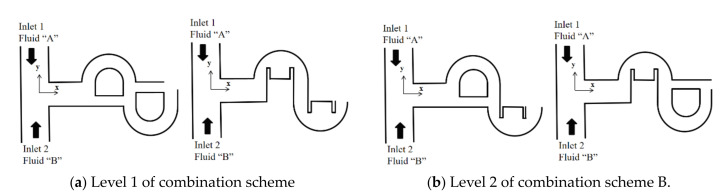
Four possible layouts of the first combination unit (nonproportional).

For each combination scheme, there were two levels, as listed in Table 1. Figure 3 shows how the layout of a combined micromixer depended on combination scheme B. Level 1 of combination scheme B meant that the second slot should be the same as the first slot. Therefore, there were two different layouts possible, depending on level of combination scheme A, as shown in Figure 3a. On the other hand, level 2 of combination scheme B meant that the second slot should be different from the first slot. Figure 3b shows two possible layouts, depending on level of the combination scheme A.

**Figure 4:**
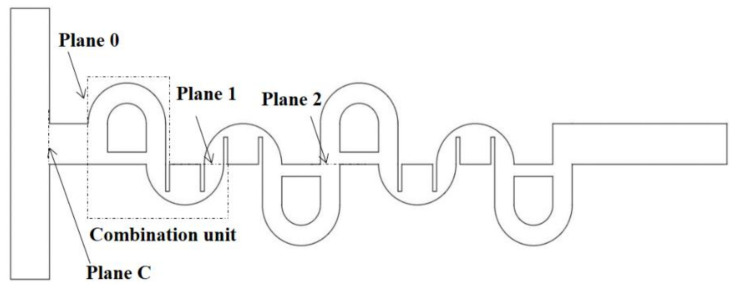
A micromixer created using four combination schemes.

Figure 4 shows a schematic diagram of a combined micromixer constructed with four CC-SARs and four MC-Bs. It was constructed, choosing level 1 for (A), level 2 for (B), level 2 for (C), and level 1 for (D). Planes 1 and 2 in the figure indicate the corresponding cross-section after the first and second combination units, respectively. Plane 0 indicates the cross-section just before the first combination unit, and plane C is the cross–section at the confluence of two streams.

## 3. Governing Equations and Computational Procedure

The fluid was assumed to be Newtonian and incompressible, and the following continuity and Navier–Stokes equations were used as the governing equations:

(1) $$\left(\vec{u}\cdot \nabla \right)\vec{u}=-\frac{1}{\rho }\nabla p+\nu \nabla^{2}\vec{u}$$

(2) $$\nabla \cdot \vec{u}=0$$

where $\vec{u}$, *p*, and ν are the velocity vector, pressure, and kinematic viscosity, respectively. The evolution of mixing was simulated by solving an advection-diffusion equation:

(3) $$\left(\vec{u}\cdot \nabla \right)\varphi =D\nabla^{2}\varphi$$

where *D* and *φ* are the diffusion coefficient and mass fraction of fluid A, respectively.

The governing Equations (1)–(3) were solved using commercial software, FLUENT 19.2 [21], which is based on the finite volume method. The QUICK scheme (quadratic upstream interpolation for convective kinematics) was used to discretize the convective terms in Equations (1) and (3), and its theoretical accuracy was third order. The velocity distribution at the two inlets was assumed to be uniform, while the outflow condition was specified at the outlet. All other walls were treated as a no-slip boundary. The mass fraction of fluid A was specified *φ* = 1 at inlet 1, and *φ* = 0 at inlet 2.

In order to obtain a fully converged solution, the computation was continued until the residuals of all equations showed negligible change after iterations—this took about 4000 iterations, on average. The corresponding residuals of continuity, momentum, and concentration equations were typically about 10^−12^, 10^−14^, and 10^−9^, respectively.

The mixing performance of the combined micromixer was evaluated using degree of mixing (DOM) and mixing energy cost (MEC). The DOM is defined in the following form:

(4) $$DOM=1-\frac{1}{\xi }\sqrt{\sum_{i=1}^{n}\frac{{\left(\phi_{i}-\xi \right)}^{2}}{n}}$$

where *φ_ι_* and *n* are the mass fraction of fluid A in the *i*th cell and the total number of cells, respectively. ξ = 0.5, which means equal mixing of the two fluids. The MEC was used to evaluate the effectiveness of a micromixer and was defined by combining the pressure load and DOM in the following form [23,24]:

(5) MEC=Δpρumean2DOM✕100

where $u_{mean}$ is the average velocity at the outlet, and Δp is the pressure load between the inlet and outlet.

The aqueous fluid flows into the two inlets were assumed to be the same. The fluid properties were assumed to be the same as the physical properties of the water. The density, diffusion constant and viscosity of the fluid were *ρ* = 997 kg/m^3^, *D* = 1.2 × 10^−9^ m^2^s^−1^, and *ν* = 0.89 × 10^−6^ m^2^s^−1^, respectively. Therefore, the Schmidt (Sc) number was approximately 10^3^ (the ratio of the kinetic viscosity and the mass diffusivity of the fluid). The Reynolds number was defined as $Re=\frac{\rho U_{mean}d_{h}}{\mu }$, where $\rho ,\;\;U_{mean},\;\;d_{h},\;\mathrm{and}\;\mu$ denote the density, the mean velocity at the outlet, the hydraulic diameter of the outlet channel, and the dynamic viscosity of the fluid, respectively.

For high Sc number simulations, the numerical diffusion may deteriorate the accuracy of the simulated results, in general. To obtain a quantitatively rigorous numerical solution, we could have used either a particle-based simulation such as the Monte Carlo method [25] or a grid-based method with a small cell Peclet number. Here, the cell Peclet number is defined as $Pe=\frac{U_{cell}l_{cell}}{D}$ where $U_{cell}$ and $l_{cell}$ are the local flow velocity and cell size, respectively. However, these are computationally expensive to adopt in an optimization study. As a remedy, some researchers proposed to artificially increase the diffusion coefficient [26]. However, most numerical studies prefer a detailed study of grid independence to obtain an acceptable agreement with the experiment [12,15,18]. It was more practical to obtain a numerical solution with a reasonable degree of accuracy, and also appropriate for the purposes of the present study.

## 4. Validation of the Numerical Study

**Figure 5:**
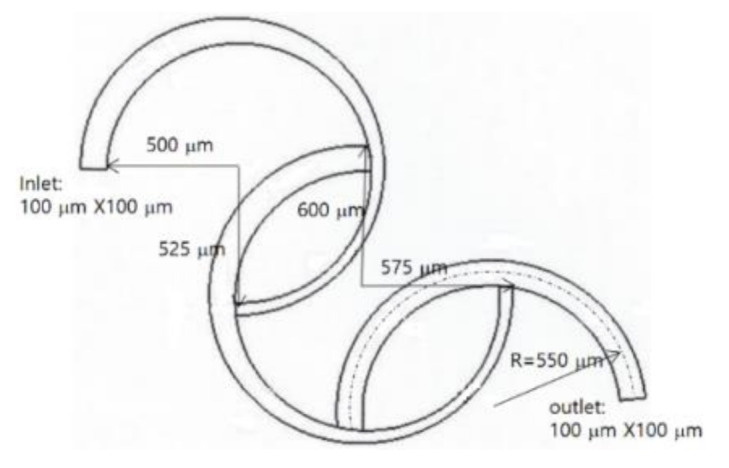
Diagram of the SAR examined by Sheu et al. [27].

The present numerical approach was validated by simulating the micromixer examined by Sheu et al. [27]. Figure 5 presents a schematic diagram consisting of three circular channels. The second and third channels were connected 180° away from the beginning of the first and second channels, respectively. The first and second circular channels were three-quarters long, and their cross-sections were rectangular. The width tapered from 100 μm to 50 μm while the depth was kept constant at 100 μm. The third channel was 180° long and its cross-section was a square 100 μm in length. The radius of curvature of all three circular channels was 550 μm.

Sheu et al. [27] defined the mixing index (MI) in the following form to evaluate mixing performance:

(6) $$MI=1-\frac{\sigma_{D}}{\sigma_{D,o}}$$

(7) $$\sigma =\sqrt{\frac{1}{n}\sum_{i=1}^{n}{\left(\varphi_{i}-\varphi_{ave}\right)}^{2}}$$

where $\sigma_{D}$ is the standard deviation of the concentration at a sampled section; $\sigma_{D,o}$ is the standard deviation at the inlet cross-section, and $\varphi_{ave}\;$ is the average concentration of fluid A at a sampled section.

Structured hexahedral cells were used to mesh the computational domain shown in Figure 5; the total number of cells is 1.75 million. The simulation was carried out and compared with the corresponding experimental data for Reynolds numbers Re = 0.5, 1, 5, 10, 25, and 50.

**Figure 6:**
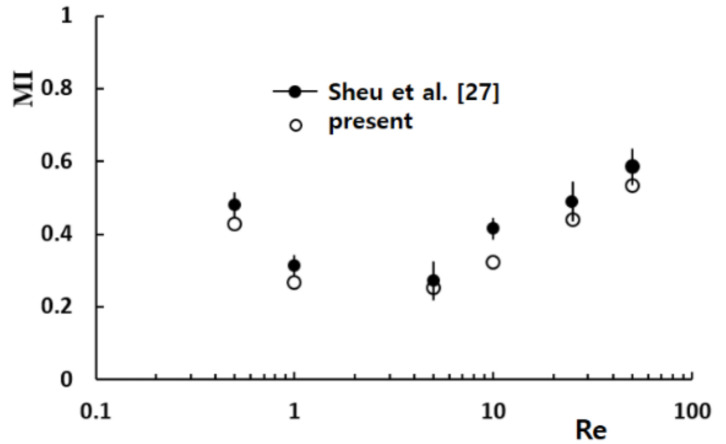
Validation of the numerical approach [27].

Figure 6 compares the present simulation results with the corresponding experimental data reported by Sheu et al. [27]. The present simulation predicted the variation of MI with the Reynolds number, and the discrepancy between the simulation results and the experimental data was acceptable. The discrepancy was attributed to several factors, including numerical diffusion and experimental uncertainty.

**Figure 7:**
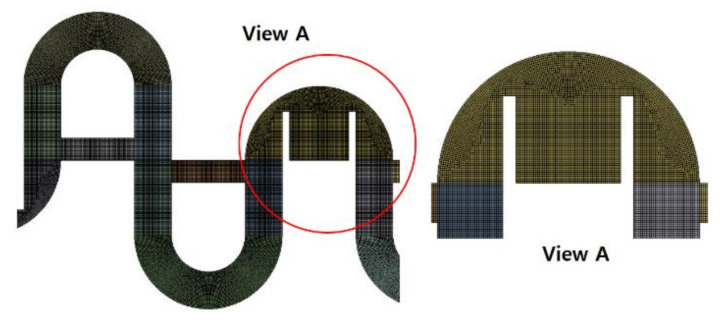
Enlarged frontal view of grid.

For the combined micromixers used in this paper, the grid independence of numerical simulations was also studied, and the micromixer depicted in Figure 4 was chosen to simulate for *Re* = 0.5. The grid independence test was carried out by varying the edge size of every cell from 4 μm to 6 μm. Figure 7 shows a typical example of grid around the two mixing units; all the grids in the outside of the MC-B are structured. The edge size of all the cells was limited to less than or equal to a specified value; it was varied from 4 μm to 6 μm for the grid independence test.

**Figure 8:**
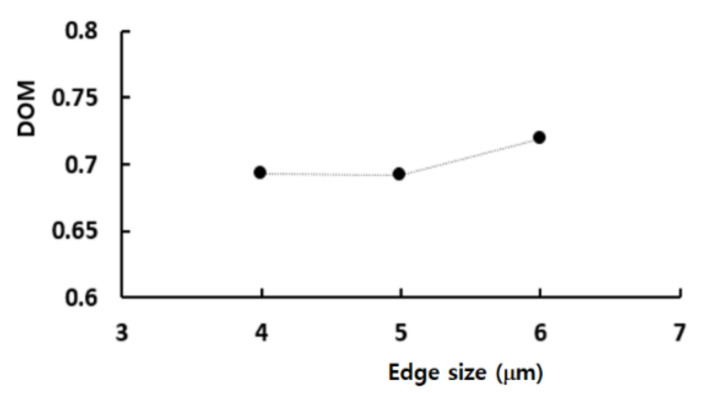
Grid independence test.

Figure 8 shows the simulated DOM for the edge size from 4 μm to 6 μm. The DOM decreased with the edge size of the computational cell, as expected. This behavior was attributed mainly to reduction of the numerical diffusion. The deviations of the 5 μm and 6 μm solutions from the 4 μm solution were 0.1% and 4%, respectively. Therefore, 5 μm was determined to be small enough to obtain solutions with reasonable accuracy.

Using the numerical solutions, the grid convergence index (GCI) was calculated to quantify the uncertainty of the grid convergence [28,29]. According to the Richardson extrapolation methodology, the GCI is calculated as follows:

(8) $$GCI=F_{s}\frac{\left|\varepsilon \right|}{r^{p}-1}$$

(9) $$\varepsilon =\frac{f_{coarse}-f_{fine}}{f_{fine}}$$

where *F_s_*, *r*, and *p* are the safety factor of the method, grid refinement ratio, and the order of accuracy of the numerical method, respectively. *f_coarse_* and *f_fine_* are the numerical results obtained with a coarse grid and fine grid, respectively. *F_s_* was specified as 1.25, as suggested by Roache [28]. For the edge sizes of 4 μm, 5 μm, and 6 μm, the corresponding number of nodes were 4.76 × 10^6^, 2.54 × 10^6^, and 1.24 × 10^6^, respectively. As a result, the GCI of the computed DOM was reduced from 10.9% to 0.25%. Therefore, an edge size of 5 μm was small enough to obtain numerical solutions with reasonable accuracy. Table 2 summarizes the result of GCI analysis.

**Figure 9:**
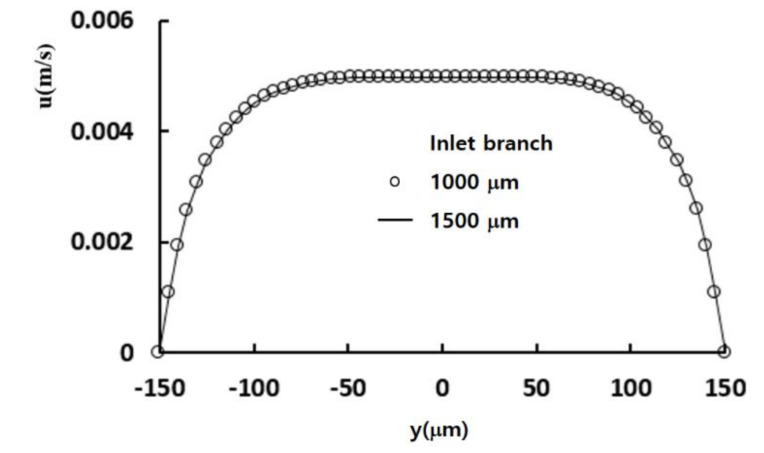
Comparison of velocity component at the confluence.

To evaluate the effects of inlet boundary conditions on the mixing performance, an additional set of computations were carried out for Re = 0.5. The DOM at the cross-section of the confluence of the two streams (plane C in Figure 4) was calculated for two different lengths of the inlet branch: 1000 μm and 1500 μm. The simulated DOMs were 0.041399 and 0.041379 for 1000 μm and 1500 μm, respectively. The difference between them was about 0.05%, and the assumption of uniform velocity at the inlet boundary had negligible effects on the mixing performance. Figure 9 shows a comparison of the velocity component, in the x-direction, along the center line at the cross section of the confluence and along the line of mid-width at the plane C in Figure 4. It also confirmed that the inlet branch of 1000 μm was long enough to neglect the effects of uniform velocity assumption at the inlet boundary on the micromixer flow.

## 5. Results and Discussion

The statistical significance of the four combination schemes, (A), (B), (C), and (D), was studied using the full factorial method to generate all possible combinations of two mixing units. All possible levels of the four combination schemes are summarized in Table 1. For example: either 1 or 2 could be chosen for combination scheme (A). The full factorial method resulted in sixteen combined micromixers, as listed in Table 3. For example: combination #4 was constructed by choosing level 1 for (A), level 2 for (B), level 2 for (C), and level 1 for (D). All combined micromixers listed in Table 3 were simulated for Re = 0.5, 2, and 50 in order to evaluate the statistical significance of the four combination schemes.

**Figure 10:**
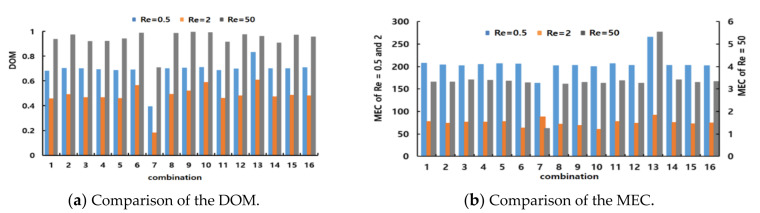
Comparison of the mixing performance of the 16 combinations.

Figure 10 presents the simulated DOM and MEC variations of all combinations for the three Reynolds numbers and shows two distinct features of the mixing performances of micromixers. First, the MEC decreased with increasing Reynolds numbers, suggesting that mixing was less cost-effective as the Reynolds number decreased. Second, mixing was performed least in the transition regime for all combinations, as shown in Figure 10a; Re = 2 was in the transition regime. Among the sixteen combinations, micromixers #7 and #13 were constructed with only one mixing unit (CC-SAR and MC-B, respectively, which were two degenerated cases of the combination schemes). Therefore, they exhibited different behaviors, distinct from those of the others. Micromixer #7 showed the lowest DOM for all three Reynolds numbers, while MEC was best for Re = 50. This suggests that the CC-SAR was effective only in the advection dominance regime. In contrast, micromixer #13 showed the highest value of MEC for Re = 0.5; the MC-B was more effective in the molecular diffusion dominance regime. These two degenerated cases showed that the two mixing units had very different characteristics. The mixing performance of all other combined micromixers was in between these two extreme cases, suggesting that an optimal combination exists in terms of the DOM and MEC.

**Figure 11:**
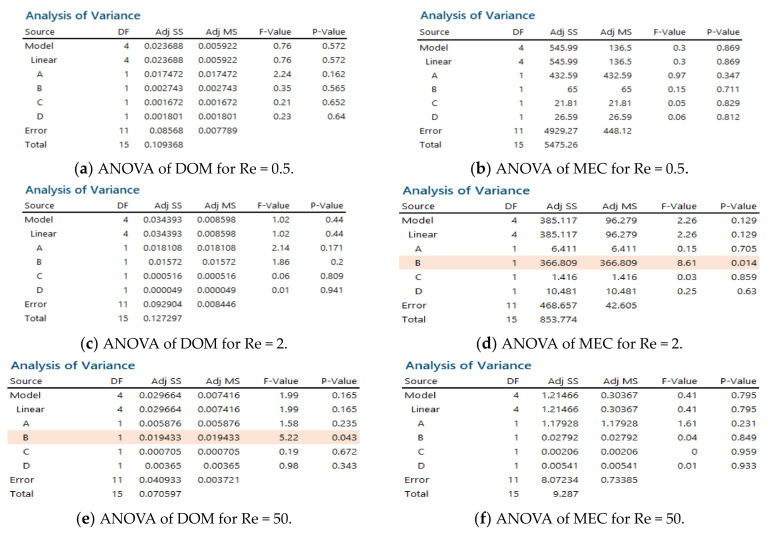
Statistical analysis of the four combination schemes.

The mixing performance was analyzed further using analysis of variance (ANOVA) to evaluate the statistical significance of the four combination schemes; Minitab 19 [30] was used for ANOVA. Figure 11 summarizes the results for Re = 0.5, 2, and 50. Here, Adj SS stands for the adjusted sum of squares, and represents a measure of variation from the mean. Adj MS indicates the mean squares, which represents an estimate of the population variance. This was calculated by dividing the corresponding sum of squares by the degrees of freedom. The F-value is the test statistic used to determine if the term is associated with the response. The *p*-value is the probability that measures the evidence against the null hypothesis. When the *p* < 0.05, the corresponding factor was statistically significant; *p* < 0.05 guarantees a 95% probability of statistical significance. The combination scheme (B) had significant effects on mixing for Re = 2 and 50, *p* = 0.014 for Re = 2 and *p* = 0.043 for Re = 50. This suggested that mixing in the regime of molecular diffusion dominance was affected negligibly by the combination order of mixing units. In contrast, it played a significant role the mixing regime of transition and advection dominance.

Combination scheme (B), which had different mixing units in the first combination unit, was statistically significant. It was generalized by applying it to other combination units (2, 3, and 4). For this study, the full factorial method was used to generate all possible designs of the four combination units. For each combination unit, either 1 (same mixing unit: two CC-SAR and two MC-B), or 2 (different mixing units: (CC-SAR and MC-B) and (MC-B and CC-SAR)) were chosen. The 16 micromixer designs listed in Table 4 are the results of the full factorial method. For example, combination #9 resulted when level 2 was chosen for all four combination units. It was obtained by applying the general combination scheme in every two consecutive mixing slots; its mixing performance was expected to be the best among the sixteen combinations. Additional simulations were carried out to evaluate the statistical significance of the general combination scheme of the two mixing units. The simulations were made for Re = 2 and 50; each represented the mixing regime of transition or advection dominance, respectively.

**Figure 12:**
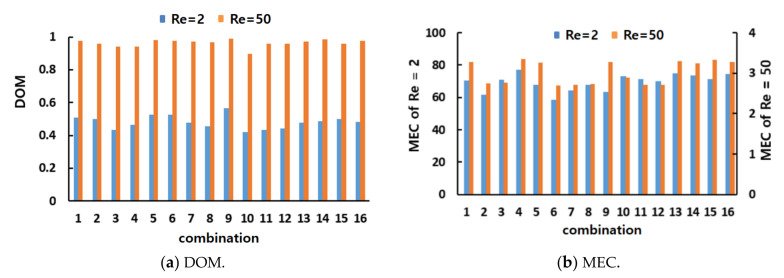
Comparison of the mixing performance of the sixteen combinations.

Figure 12 compares the simulated DOM and MEC of all sixteen combinations for Re = 2 and 50. As expected, combination #9 showed the largest DOM for Re = 2 and 50. Hence, the general combination scheme, i.e., different mixing units in any two consecutive mixing slots, was the optimal combination of the CC-SARs and MC-Bs.

**Figure 13:**
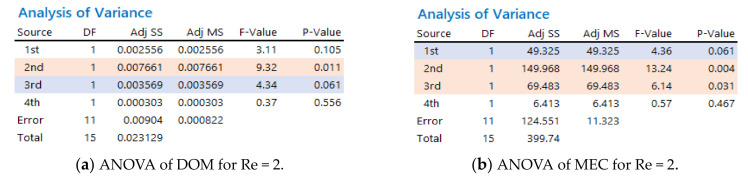

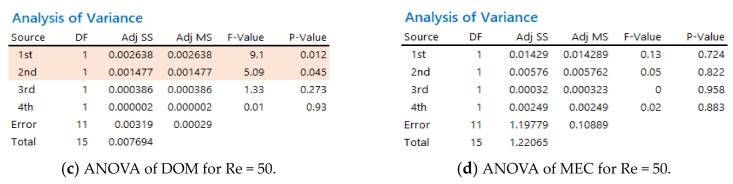
Statistical analysis of the combination scheme (B) for four combination units.

The statistical significance of the general combination scheme was analyzed further using ANOVA. Figure 13 summarizes the ANOVA results of the general combination scheme based on the mixing performance of the DOM and MEC. For Re = 2, the general combination scheme was significant in terms of the MEC for the first, second, and third combination units; the probability of significance for the first mixing unit was approximately 94%. This was also significant in terms of the DOM for the second and third combination units; the probability of significance for the third mixing unit was approximately 94%. For Re = 50, the general combination scheme was significant in terms of the DOM for the first and second combination units. This result confirmed that the general combination scheme plays a significant role in the mixing performance of a combined micromixer, particularly in the mixing regime of transition and advection dominance. On the other hand, a combination order has limited effects on the mixing performance after the third combination unit.

An additional set of simulations was carried out to examine how the general combination scheme affects the mixing performance of the combined micromixers for a wide range of the Reynolds numbers. The combinations #2, #7, #8, and #9 were simulated for Reynolds numbers ranging from 0.5 to 80. Combinations #2, #7, and #8 did not adopt the general combination scheme in the first, third, and second mixing units, respectively. In contrast, combination #9 adopted the general combination scheme throughout the micromixer (different mixing units in arbitrary consecutive mixing slots).

**Figure 14:**
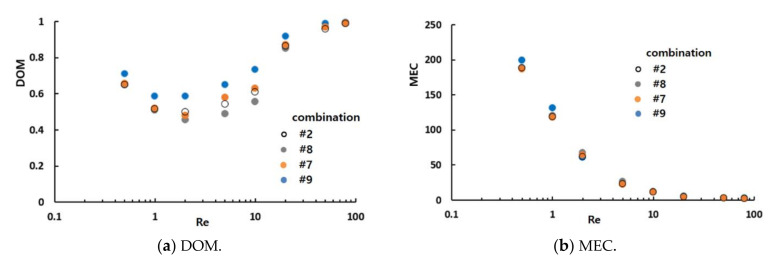
Comparison of the mixing performance of the four combinations.

Figure 14 compares the mixing performances of the four combinations (#2, #7, #8, and #9) in terms of the DOM and MEC. The DOM of combination #9 showed noticeable improvement over the entire range of Reynolds numbers. For example, the DOM of #9 was enhanced approximately 18%, 23%, and 29% from that of combinations #2, #7, and #8, respectively, for Re = 2. This enhancement confirmed that the general combination scheme, which had different mixing units in any two consecutive mixing slots, should be a major design parameter in combining different mixing units. Figure 14b compares the MEC of the four combinations. Combination #9 was the most cost-effective over a wide range of Reynolds numbers.

**Figure 15:**
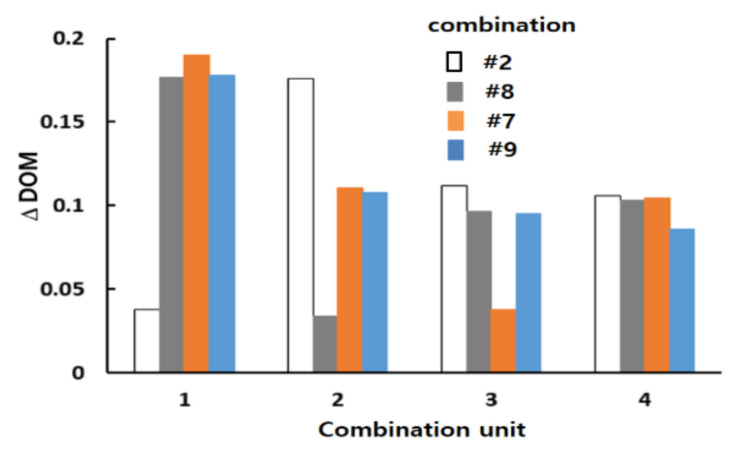
Comparison of the DOM increment in each combination unit for Re = 2.

Figure 15 shows how the general combination scheme affected the DOM enhancement in the corresponding combination unit (the DOM increment in each combination unit of the four combinations for Re = 2). For example, the DOM increment in the first combination unit was calculated by the difference in the DOM between planes 0 and 1 in Figure 4. Compared to the increase in combination #9, the other combinations each showed a smaller increase in one of the four combination units. For example, the increase in the first combination unit of combination #2 was smaller than other combinations. Combination #2 did not adopt the general combination scheme in the first combination unit. Similarly, combinations #8 and #7 showed smaller increments in the second and third combination units, respectively.

**Figure 16:**
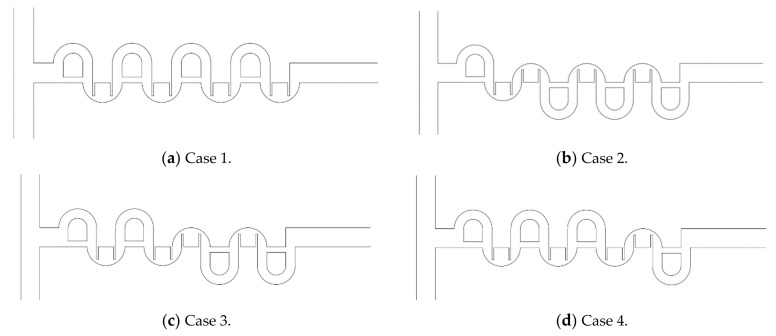
Schematic diagram of four micromixers combined with four CC-SARs and four MC-Bs.

The general combination scheme was assessed by comparing the mixing performances of the four different combinations based on the same number of mixing units. Figure 16 shows the four combinations that adopted the combination scheme in every combination unit (different mixing units in each combination unit). On the other hand, cases 2, 3, and 4 did not adopt the general combination scheme completely; it was not adopted at one junction of the combination units. The mixing unit (MC-B) was repeated in the second and third mixing slots of case 2, even though it used different mixing units in all combination units. On the other hand, case 1 adopted the general combination scheme throughout the whole mixer; different mixing units were used in any two consecutive mixing slots.

**Figure 17:**
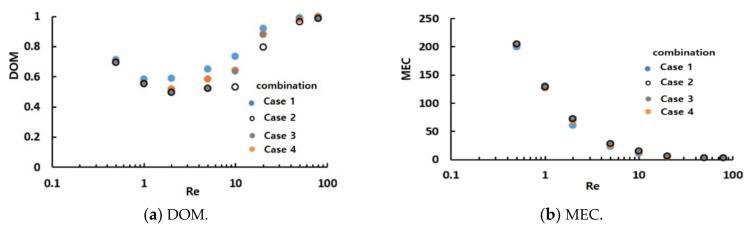
Comparison of the mixing performance of the four combinations.

**Figure 18:**
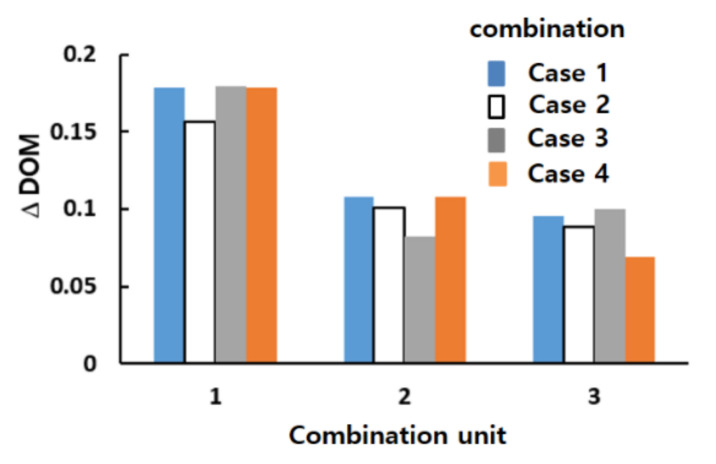
Comparison of the DOM increment in each combination unit for Re = 2.

Figure 17 compares the mixing performance of the four different combinations in terms of the DOM and MEC. As expected, case 1 showed the best DOM throughout the entire range of Reynolds numbers. The enhancement of case 1 from the other combinations was noticeable, particularly in the range of Re = 1 to Re = 20. This suggested that the general combination scheme played a significant role in the mixing regime of transition; it also played a role in the mixing regime of advection dominance. In contrast, the effects of a combination order were negligible in the mixing regime of molecular dominance; the Reynolds number was less than or equal to approximately 1. Figure 18 compares the DOM increments of the four cases in each combination unit for Re = 2. Cases 2, 3, and 4 showed a smaller increase in DOM in the combination unit joined to the next combination unit without adopting the general combination scheme. This result confirmed that the general combination scheme should be adopted to enhance the mixing performance at the junction of two combination units and in each combination unit.

**Figure 19:**
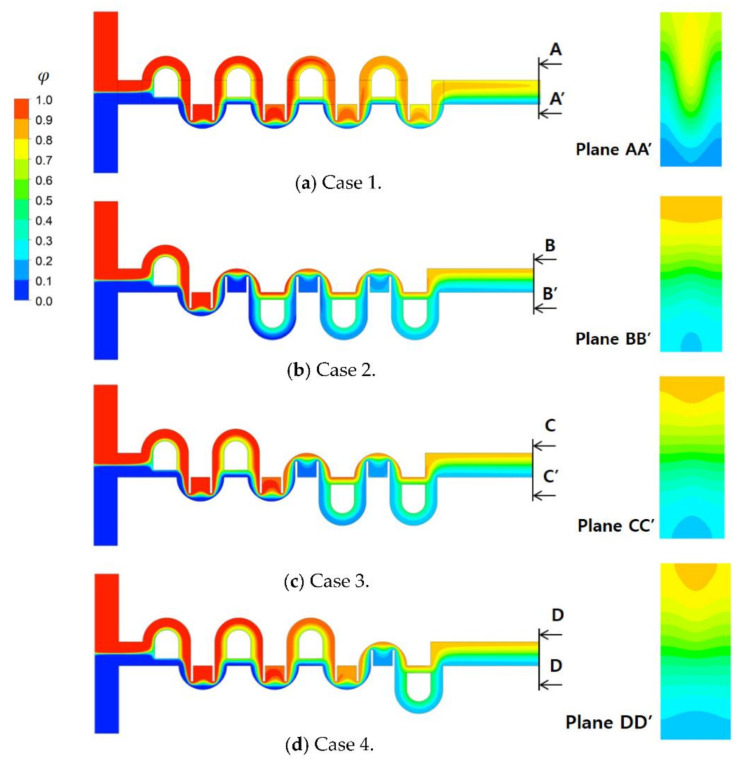
Comparison of the mass concentration distribution of the four cases for Re = 2.

Figure 19 compares the mass concentration distribution of the four combined micromixers at the plane of the mid-width, z = 60 μm, and at the outlet plane. Although they combined the same number of mixing units, the evolution of mixing and the mass concentration of fluid A at the outlet plane were entirely dependent on how they were combined. Because case 1 adopted the general combination scheme throughout the entire micromixer, its mass concentration of fluid A at the outlet plane showed the most mixed distribution, compared to the other cases. This confirmed the significance of the general combination scheme in a combined micromixer.

## 6. Conclusions

This study examined how the combination order of mixing units affected the mixing performance of a combined micromixer. Passive micromixers were constructed using two mixing units: cross-channel SAR (CC-SAR) and mixing cell with baffles (MC-B). The four combination schemes were proposed to construct a combined micromixer using two mixing units. The micromixer consisted of eight mixing slots and two consecutive mixing slots grouped as a combination unit; the micromixer had four combination units.

Sixteen combined micromixers were constructed according to the four combination schemes in order to study the effects of the combination schemes on mixing performance. Their mixing performance was simulated using commercial software, FLUENT 19.2 MEC, for three Reynolds numbers (Re = 0.5, 2, and 50). Each Reynolds number represented a mixing regime in which the dominant mixing mechanism was different. Re = 0.5 represented the mixing regime where molecular diffusion was dominant. Re = 50 represented for a mixing regime with chaotic advection dominance. Re = 2 represented the transition regime where mixing due to molecular diffusion and chaotic advection were of equal significance. The simulation results were analyzed to evaluate the statistical significance of the four combination schemes using ANOVA, regarding the DOM and MEC.

Among the four combination schemes, combination scheme (B) was statistically significant for Re = 2 and 50 (different mixing units in the first combination unit). None of the four combination schemes were significant for Re = 0.5. The mixing performance of the combined micromixer was greatly affected by the combination scheme when operated in the mixing regimes of transition or chaotic advection dominance. In contrast, the effects of the combination scheme were negligible in the mixing regime of molecular diffusion dominance.

The significance of the combination scheme (B) was generalized by applying it to other subsequent combination units; combining the different mixing units in any two consecutive mixing slots was defined as the general combination scheme. The general combination scheme was evaluated by simulating sixteen combined micromixers for Reynolds numbers Re = 2 and 50. For Re = 2, the general combination scheme was statistically significant in the first, second, and third combination units. For Re = 50, it was statistically significant in the first and second combination units. The general combination scheme played a significant role in the mixing performance of a combined micromixer, particularly in the mixing regime of transition and advection dominance. On the other hand, the combination order had limited effects on the mixing performance after three combination units.

A micromixer based on the general combination scheme showed the best performance over a wide range of Reynolds numbers, compared to other micromixers with the same number of mixing units. The enhancement was most prominent in the mixing regime of the transition, and it was negligible in the mixing regime of the molecular dominance.

## Figures and Tables

**Table 1:** Four combination schemes (A, B, C, and D) and their levels.

Design Variable (Combination Scheme)	Levels	
	1	2
A (slot 1)	CC-SAR	MC-B
B (combination unit 1)	same mixing units	different mixing units
C (combination module 1)	same combination units	reversed combination units
D (combination module 2)	same combination modules	reversed combination modules

**Table 2:** Summary of GCI analysis.

Edge Size (μm)	No. of Nodes	r	DOM	GCI
4	4.76 × 10^6^	1.25	0.693001	0.00246
5	2.54 × 10^6^	1.2	0.692234	0.0109
6	1.24 × 10^6^		0.718825	-

**Table 3:** Full factorial combination for eight slots.

Combination	From Slot 1 to Slot 8			
	(A)	(B)	(C)	(D)
#1	(CC-SAR)	(CC-SAR)	(CC-SAR) + (CC-SAR)	(MC-B) + (MC-B) + (MC-B) + (MC-B)
#2	(MC-B)	(CC-SAR)	(CC-SAR) + (MC-B)	(MC-B) + (CC-SAR) + (CC-SAR) + (MC-B)
#3	(MC-B)	(MC-B)	(CC-SAR) + (CC-SAR)	(MC-B) + (MC-B) + (CC-SAR) + (CC-SAR)
#4	(CC-SAR)	(MC-B)	(MC-B) + (CC-SAR)	(CC-SAR) + (MC-B) + (MC-B) + (CC-SAR)
#5	(CC-SAR)	(CC-SAR)	(MC-B) + (MC-B)	(CC-SAR) + (CC-SAR) + (MC-B) + (MC-B)
#6	(CC-SAR)	(MC-B)	(CC-SAR) + (MC-B)	(CC-SAR) + (MC-B) + (CC-SAR) + (MC-B)
#7	(CC-SAR)	(CC-SAR)	(CC-SAR) + (CC-SAR)	(CC-SAR) + (CC-SAR) + (CC-SAR) + (CC-SAR)
#8	(CC-SAR)	(MC-B)	(CC-SAR) + (MC-B)	(MC-B) + (CC-SAR) + (MC-B) + (CC-SAR)
#9	(MC-B)	(CC-SAR)	(MC-B) + (CC-SAR)	(CC-SAR) + (MC-B) + (CC-SAR) + (MC-B)
#10	(MC-B)	(CC-SAR)	(MC-B) + (CC-SAR)	(MC-B) + (CC-SAR) + (MC-B) + (CC-SAR)
#11	(CC-SAR)	(CC-SAR)	(MC-B) + (MC-B)	(MC-B) + (MC-B) + (CC-SAR) + (CC-SAR)
#12	(MC-B)	(CC-SAR)	(CC-SAR) + (MC-B)	(CC-SAR) + (MC-B) + (MC-B) + (CC-SAR)
#13	(MC-B)	(MC-B)	(MC-B) + (MC-B)	(MC-B) + (MC-B) + (MC-B) + (MC-B)
#14	(MC-B)	(MC-B)	(MC-B) + (MC-B)	(CC-SAR) + (CC-SAR) + (CC-SAR) + (CC-SAR)
#15	(CC-SAR)	(MC-B)	(MC-B) + (CC-SAR)	(MC-B) + (CC-SAR) + (CC-SAR) + (MC-B)
#16	(MC-B)	(MC-B)	(CC-SAR) + (CC-SAR)	(CC-SAR) + (CC-SAR) + (MC-B) + (MC-B)

**Table 4:** Full factorial combination for the four combination units.

Combination	From Combination Unit 1 to Unit 4			
	1st Unit	2nd Unit	3rd Unit	4th Unit
#1	(CC-SAR) + (CC-SAR)	(MC-B) + (CC-SAR)	(MC-B) + (CC-SAR)	(MC-B) + (MC-B)
#2	(CC-SAR) + (CC-SAR)	(MC-B) + (CC-SAR)	(MC-B) + (CC-SAR)	(MC-B) + (CC-SAR)
#3	(CC-SAR) + (CC-SAR)	(MC-B) + (MC-B)	(CC-SAR) + (MC-B)	(CC-SAR) + (CC-SAR)
#4	(CC-SAR) + (CC-SAR)	(MC-B) + (MC-B)	(CC-SAR) + (CC-SAR)	(MC-B) + (MC-B)
#5	(CC-SAR) + (MC-B)	(CC-SAR) + (MC-B)	(CC-SAR) + (CC-SAR)	(MC-B) + (MC-B)
#6	(CC-SAR) + (MC-B)	(CC-SAR) + (MC-B)	(CC-SAR) + (MC-B)	(CC-SAR) + (CC-SAR)
#7	(CC-SAR) + (MC-B)	(CC-SAR) + (MC-B)	(CC-SAR) + (CC-SAR)	(MC-B) + (CC-SAR)
#8	(CC-SAR) + (MC-B)	(CC-SAR) + (CC-SAR)	(MC-B) + (CC-SAR)	(MC-B) + (CC-SAR)
#9	(CC-SAR) + (MC-B)	(CC-SAR) + (MC-B)	(CC-SAR) + (MC-B)	(CC-SAR) + (MC-B)
#10	(CC-SAR) + (CC-SAR)	(MC-B) + (MC-B)	(CC-SAR) + (CC-SAR)	(MC-B) + (CC-SAR)
#11	(CC-SAR) + (MC-B)	(CC-SAR) + (CC-SAR)	(MC-B) + (MC-B)	(CC-SAR) + (CC-SAR)
#12	(CC-SAR) + (CC-SAR)	(MC-B) + (CC-SAR)	(MC-B) + (MC-B)	(CC-SAR) + (CC-SAR)
#13	(CC-SAR) + (CC-SAR)	(MC-B) + (CC-SAR)	(MC-B) + (MC-B)	(CC-SAR) + (MC-B)
#14	(CC-SAR) + (MC-B)	(CC-SAR) + (CC-SAR)	(MC-B) + (MC-B)	(CC-SAR) + (MC-B)
#15	(CC-SAR) + (CC-SAR)	(MC-B) + (MC-B)	(CC-SAR) + (MC-B)	(CC-SAR) + (MC-B)
#16	(CC-SAR) + (MC-B)	(CC-SAR) + (CC-SAR)	(MC-B) + (CC-SAR)	(MC-B) + (MC-B)

## Nomenclature

*CC-SAR*	cross channel-split and recombine
*D*	diffusion coefficient (m^2^/s)
*DOM*	degree of mixing
*f_coarse_*	numerical solution obtained with a coarse grid
*f_fine_*	numerical solution obtained with a fine grid
*GCI*	grid convergence index
*H*	height of baffle (μm)
*MC-B*	mixing chamber with baffles
*MEC*	mixing energy cost
*MI*	mixing index
*n*	total number of sampling points at a specific plane
Δp	pressure load (Pa)
*r*	grid refinement ratio
*Re*	Reynolds number
*U_mean_*	average velocity at the outlet (m/s)
$\vec{u}$	velocity vector
*x, y, z*	Cartesian coordinates
**greek symbols**	
*ε*	relative error
*μ*	fluid viscosity (kg/(ms))
*ν*	fluid kinematic viscosity (m^2^/s)
*φ*	mass fraction of fluid A
*ρ*	fluid density (kg/m^3^)
*σ*	standard deviation
**subscripts**	
*i*	sampling point
